# Transvaginal versus transabdominal specimen extraction in minimally invasive surgery: a systematic review and meta-analysis

**DOI:** 10.1007/s00423-024-03361-5

**Published:** 2024-06-03

**Authors:** Jasmine Hui Er Chang, Hongyun Xu, Yun Zhao, Ian Jun Yan Wee, Joella Xiaohong Ang, Emile Kwong-Wei Tan, Isaac Seow-En

**Affiliations:** 1 Department of Colorectal Surgery, Singapore General Hospital, Outram Road, 169608 Singapore; 2 Group Finance Analytics, Singapore Health Services, Singapore, Singapore; 3 Department of Obstetrics & Gynaecology, Singapore General Hospital, Outram Road, 169608 Singapore

**Keywords:** Natural orifice specimen extraction, Transvaginal, Laparoscopic surgery, Meta-analysis

## Abstract

**Aim:**

Natural orifice specimen extraction (NOSE) is an alternative to conventional transabdominal retrieval. We aimed to compare outcomes following transvaginal specimen extraction (TVSE) and transabdominal specimen extraction (TASE) in minimally invasive abdominal surgery.

**Methods:**

An electronic database search of PubMed, Embase and CENTRAL was performed from inception until March 2023. Comparative studies evaluating TVSE versus TASE in adult female patients were included. Studies involving transanal NOSE, endoluminal surgery, or TVSE with concomitant hysterectomy were excluded. Weighted mean differences (WMD) and odds ratio were estimated for continuous and dichotomous outcomes respectively. Primary outcomes were postoperative day 1 (POD1) pain and length of stay (LOS). Secondary outcomes were operative time, rescue analgesia, morbidity, and cosmesis. A review of sexual, oncological, and technical outcomes was performed.

**Results:**

Thirteen studies (2 randomised trials, 11 retrospective cohort studies), involving 1094 patients (TASE 583, TVSE 511), were included in the analysis. Seven studies involved colorectal disease and six assessed gynaecological conditions. TVSE resulted in significantly decreased POD1 pain (WMD 1.08, 95% CI: 0.49, 1.68) and shorter LOS (WMD 1.18 days, 95% CI: 0.14, 2.22), compared to TASE. Operative time was similar between both groups, with fewer patients requiring postoperative rescue analgesia with TVSE. Overall morbidity rates, as well as both wound-related and non-wound related complication rates were better with TVSE, while anastomotic morbidity rates were comparable. Cosmetic scores were higher with TVSE. TVSE did not result in worse sexual or oncological outcomes.

**Conclusion:**

TVSE may be feasible and beneficial compared to TASE when performed by proficient laparoscopic operators, using appropriate selection criteria. Continued evaluation with prospective studies is warranted.

**Supplementary Information:**

The online version contains supplementary material available at 10.1007/s00423-024-03361-5.

## Introduction

The advent of laparoscopy has revolutionised the practice of abdominal surgery. Reduced abdominal incision size results in decreased trauma, postoperative pain and wound complications, quicker recovery, and shorter hospitalisation times. Minimally invasive techniques have become the cornerstone of enhanced recovery after surgery programmes [1] and may be approaching the standard of care even for complex abdominal procedures, including colorectal cancer surgery [2], liver resection [3], and gynaecological cancer surgery [4].

Conventional laparoscopy still requires an additional abdominal incision for specimen retrieval, commonly a mini-laparotomy or Pfannenstiel incision. Natural orifice specimen extraction (NOSE) via the anus or vagina provides a viable alternative to conventional specimen extraction. NOSE further reduces abdominal wall operative trauma and enhances the benefits of minimally invasive surgery, with proven early postoperative advantages over transabdominal specimen extraction (TASE) without compromising oncological outcomes [5–9].

The vagina has several advantages over the anus for specimen extraction, including the clean environment, lack of a high-pressure anal sphincter, and elastic walls allowing the passage of larger specimens, with a reduced risk of contamination and specimen rupture [10, 11]. In the field of gynaecological surgery, the vagina is a convenient route of extraction following laparoscopic hysterectomy. The safety and feasibility of transvaginal natural orifice transluminal endoscopic surgery (vNOTES), a novel technique used for both surgical dissection and specimen extraction, has been demonstrated for hysterectomies [12], as well as conditions without concomitant hysterectomy, including cholecystectomy, adnexectomy, and appendicectomy [13].

Interestingly, the first colonic resection with transvaginal NOSE via a colpotomy was reported in 1991 [14], the same year the first series of laparoscopic colectomies was described [15]. Nonetheless, it was almost two decades later that transvaginal specimen extraction (TVSE) following colorectal surgery was again demonstrated, for a case of right hemicolectomy [16], as well as a series of seven patients who underwent laparoscopic proctocolectomy with ileal pouch-anal anastomosis [17]. For patients with an intact uterus, the TVSE technique involves the creation of the posterior colpotomy for specimen extraction, with subsequent closure of the defect.

In recent years, increasing interest in NOSE in colorectal surgery is reflected by the abundance of meta-analyses on the subject [5–9]. To our knowledge, however, no meta-analysis has specifically compared TVSE versus TASE in minimally invasive abdominal surgery. Given the accumulating evidence, an evaluation of these methods of specimen retrieval is timely and may help to guide future practice.

## Methodology

### Literature search

A systematic search was performed in accordance with the Preferred Reporting Items for Systematic Reviews and Meta-Analyses (PRISMA) guidelines and was registered with PROSPERO (ID CRD42023439645). Two reviewers (JC, HX) independently conducted electronic database searches on PubMed, Embase and CENTRAL (Cochrane Central Register of Controlled Trials) to identify relevant articles published in the English Language Keywords related to “natural orifice specimen extraction”, “transvaginal”, “transcolonic”, “transrectal”, and “transanal”, from inception until March 2023 were searched. A manual search of the reference lists of included studies was performed to identify additional relevant articles. Grey literature was excluded. The full search strategy is presented in Appendix 1.

### Eligibility criteria

Randomised controlled trials, case-control and cohort studies comparing TVSE with TASE following laparoscopic or robotic abdominal surgery were included. The inclusion criteria were: (1) Quantitative comparative studies comparing TVSE versus TASE; (2) Studies on adult patients ≥ 18 years with benign or malignant diseases treated with laparoscopic or robotic surgery; (3) Studies using TVSE as an intervention; and (4) Studies comparing at least one postoperative outcome.

The exclusion criteria were: (1) Studies comparing transluminal or endoluminal surgical techniques; (2) Studies comparing TVSE following hysterectomy; (3) Studies on paediatric patients; (4) Non-human studies; (5) Non-English studies (6) Non-comparative studies including reviews, editorials, and letters.

### Selection of studies and data collection

Two independent reviewers (JC, HX) screened the title and abstract of each article for relevance. The full-text articles were then retrieved for further detailed review for confirmation for study inclusion. Conflicts were resolved by consensus or by appeal to the senior author (ISE).

The following information was extracted from included studies: (1) Patient characteristics including age, body mass index (BMI), American Society of Anaesthesiologists (ASA) score, history of previous surgery and co-morbidities; (2) Operative details including operative indication, type of surgery, operative time, blood loss, route of specimen extraction, conversion to open surgery, and intraoperative complications; (3) Postoperative outcomes including pain score, use of analgesia, length of hospital stay, complication rate, cosmetic outcome and sexual dysfunction. When the mean and standard deviation for continuous variables were not available, it was calculated based on the methods described by Luo et al. [18] and Wan et al. [19].

### Outcomes

The main benefit of NOSE surgery is the reduced iatrogenic abdominal wall trauma and postoperative wound pain. Diminished pain, along with the consequent decrease in opioid analgesia use, facilitates quicker mobilisation, bowel recovery, and ultimately, earlier discharge from hospital. Postoperative day 1 (POD1) pain score and hospital length of stay (LOS) were therefore selected as the primary outcome measures of this meta-analysis. Secondary outcomes were operative time, use of postoperative rescue analgesia, postoperative morbidity, and patient-reported cosmesis scores. A review of postoperative sexual dysfunction, oncological and technical outcomes was also performed.

### Data analysis

Statistical analyses were performed in R statistical software (version 4.3.1). The pooled odds ratio (OR) and its corresponding 95% confidence interval (CI) were computed for dichotomous outcomes. The calculation of the weighted mean difference (MD) with a 95% CI was performed for continuous outcomes. The aggregation of study data was performed using a random-effects model, enabling the synthesis of effect estimates.

### Risk of bias and quality assessment

Risk of bias (RoB) and quality assessment was conducted independently by two reviewers (JC, HX). Risk Of Bias In Non-randomized Studies – of Interventions (ROBINS-I) assessment tool [20] was used for retrospective cohort studies, while the Revised Cochrane risk-of-bias (RoB 2) tool [21] was used for randomised-controlled trials. Consensus was obtained, with any conflicts resolved through discussion. The results were visualised using the robvis tool [22]. Publication bias was assessed by funnel plots using Egger’s tests. The evaluation of heterogeneity was conducted using the *I*^*2*^ statistic. An *I*^*2*^ > 50% indicated significant heterogeneity.

## Results

### Study selection and characteristics

**Fig. 1:**
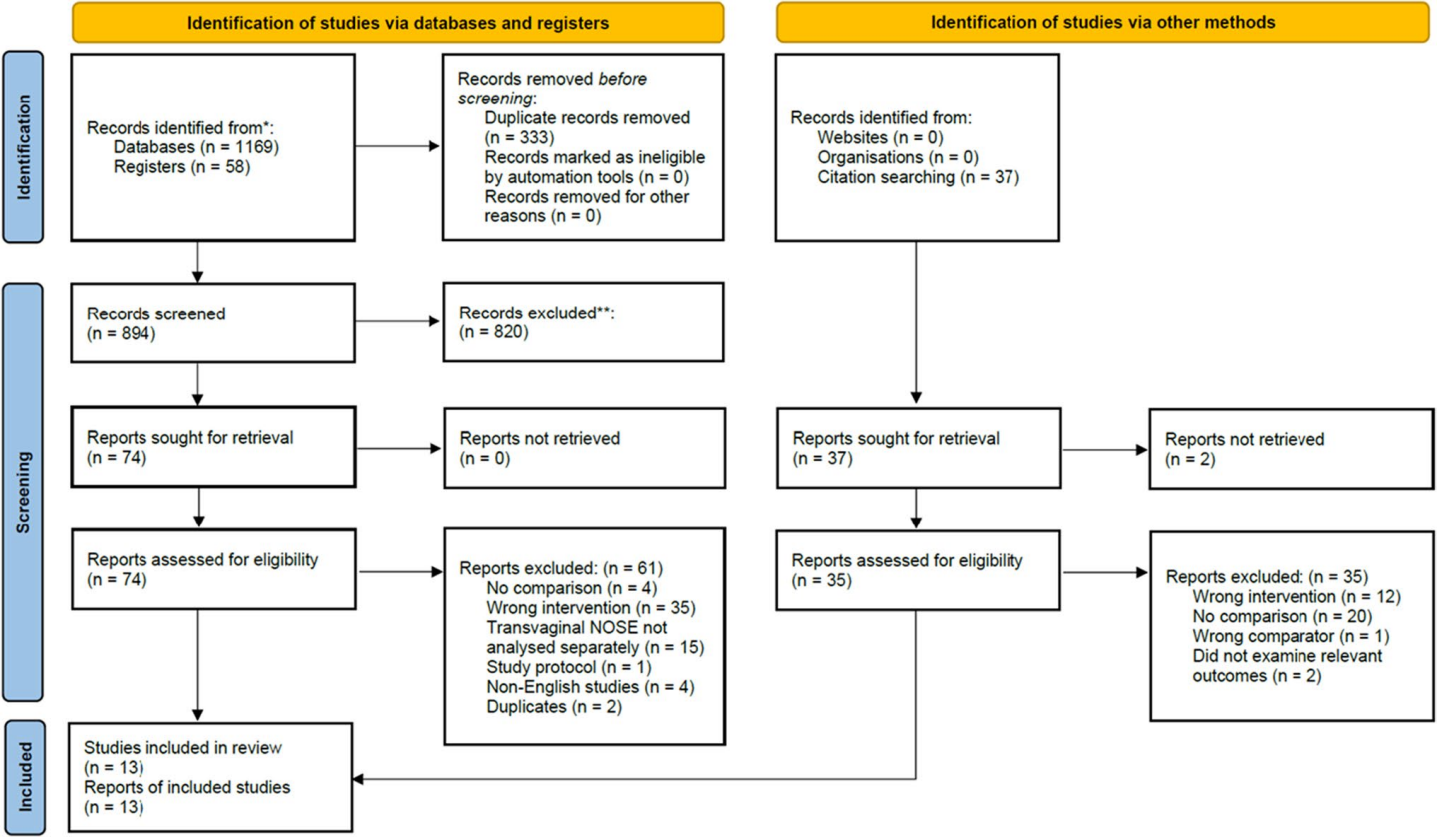
PRISMA flowchart

Database searches yielded 1227 publications. Non-relevant and duplicate publications were excluded following title and abstract review. 74 publications remained for full-text review. A PRISMA flow diagram depicts the study selection process (Fig. 1). Thirteen [23–35] comparative studies were included in the final analysis; eleven were retrospective cohort studies [23, 24, 26–31, 33–35] and two were randomised controlled trials [25, 32]. The studies were conducted in China (*n* = 4) [26–29], Turkey (*n* = 3) [30–32], Italy (*n* = 2) [24, 25], South Korea (*n* = 2) [33, 34], Spain (*n* = 1) [35], and the United States of America (*n* = 1) [23].

Three retrospective studies performed propensity-score matching [24, 27, 28]. Bogani et al. [24] estimated a propensity score based on a multivariable logistic regression model using patient age, BMI, ASA score, prior abdominal surgery or caesarean section, parity, single versus multiple myomectomies, concomitant procedures, diameter and weight of myoma(s). Li et al. [27] matched patients by age, BMI, ASA score, preoperative serum carcinoembryonic antigen level, tumour location, clinical TNM stage, and preoperative Pelvic Floor Disability Index (PFDI-20) score. Zhang et al. [28] matched patients using age, tumour diameter, location, differentiation, and TMN stage.

A total of 1094 patients were included, of which 583 (53.3%) patients underwent TASE while 511 (46.7%) underwent TVSE. Of the 13 studies, six (46.2%) assessed specimen extraction methods for benign diseases [24, 25, 30–32, 35], six (46.2%) for malignant diseases [26–29, 33, 34], and one (7.7%) study included both benign and malignant conditions [23]. Seven (53.8%) studies included colorectal disease, i.e., colorectal cancer or benign conditions [23, 26–29, 33, 34], while six (46.2%) assessed benign gynaecological disease, i.e., adnexal lesions, uterine myomas and deep endometriosis [24, 25, 30–32, 35]. Only one study by Gao et al. [26] employed a robotic-assisted approach, while all other studies performed laparoscopic surgery. Zhang et al. [28] contributed the most patients (*n* = 140) while Awad et al. [23] contributed the fewest (*n* = 40).

Table 1 summarises the characteristics of the 13 included studies.

**Table 1:** Characteristics of 13 studies comparing transvaginal specimen extraction versus transabdominal extraction in minimally invasive surgery

Author	Year	Country	Study Design	Disease type	Pathology	Patients		Age (mean ± SD)		BMI (mean ± SD)	
						TV	TA	TV	TA	TV	TA
Awad et al. [23]	2014	USA	RCS	Mixed	Right sided colonic lesions	20	20	66.9 ± 8.9	63.6 ± 9.08	25.1 ± 6.65	31.6 ± 8.33
Bogani et al. [24]	2014	Italy	RCS	Benign	Uterine myomas	50	50	36.7 ± 6.1	37.1 ± 6.4	23 (18–32) [median (range)]	24 (18–34.1) [median (range)]
Boza et al. [30]	2019	Turkey	RCS	Benign	Uterine myomas	31	31	39.5 (28-52) [median (range)]	39 (27-49) [median (range)]	23 (18-38) [median (range)]	22 (17-32) [median (range)]
Gao et al. [26]	2020	China	RCS	Malignant	Sigmoid and rectal cancers	45	45	58.1 ± 11.8	59.1 ± 10.8	22.1 ± 2.7	21.6 ± 2.2
Ghezzi et al. [25]	2012	Italy	RCT	Benign	Benign adnexal masses	34	32	47.7 ± 12.9	42.5 ± 12.5	23.8 ± 3.6	24.1 ± 3.9
Güngördük et al. [31]	2022	Turkey	RCS	Benign	Benign adnexal masses	58	35	41.4 ± 8.5	44.0 ± 10.6	30.0 ± 4.7	31.3 ± 3.7
Kim et al. [33]	2014	South Korea	RCS	Malignant	Left sided colorectal cancers	58	58	62.8 ± 9.0	63.2 ± 10.7	23.5 ± 2.9	23.2 ± 3.3
Li et al. [27]	2019	China	RCS	Malignant	Right sided colon cancers	31	31	70.06 ± 9.29	68.90 ± 11.96	23.77 ± 3.91	25.58 ± 4.70
Park et al. [34]	2011	South Korea	RCS	Malignant	Right sided colon cancers	34	34	61.0 ± 11.2	63.6 ± 11.6	23.9 ± 3.1	23.1 ± 2.7
Soyman et al. [32]	2019	Turkey	RCT	Benign	Benign adnexal masses	28	30	34.2 ± 8.3	37 ± 10.2	22.8 ± 4.7	24.1 ± 5.6
Spagnolo et al. [35]	2022	Spain	RCS	Benign	Deep endometriosis	23	76	38.00 ± 6.52	36.58 ± 4.97	23.00 ± 2.80	23.30 ± 4.75
Zhang et al. [28]	2022	China	RCS	Malignant	Left sided colorectal cancers	70	70	59.5 (31–84) [median (range)]	62.5 (30–80) [median (range)]	22.9 ± 3.09	23.2 ± 3.21
Zheng et al. [29]	2022	China	RCS	Malignant	Sigmoid and rectal cancers	52	48	≤60yo: 33 (63.46%); >60yo: 19 (36.54%)	≤60yo: 30 (62.5%); >60yo: 18 (37.5%)	≤23: 28 (53.85%); >23: 24 (46.15%)	≤23: 25 (52.08%); >23: 23 (47.92%)

*TV* transvaginal specimen extraction, *TA* transabdominal specimen extraction, *RCS* retrospective cohort study, *RCT* randomised controlled trial

### Patient selection

Twelve studies [23–29, 31–35] stated inclusion and exclusion criteria, with nine reporting specific exclusion criteria for transvaginal extraction [23–26, 28, 31–34]. Exclusion criteria for TVSE included virgo intacta [24–26, 28, 32, 34], childbearing age [26, 33], vaginal abnormalities such as narrowing, stenosis, malformation or previous infection [23, 28, 33, 34], previous pelvic surgery [34], pelvic adhesions [23, 33, 34], pelvic inflammatory disease [26, 33], history of endometriosis [23–25, 31–33], inability to assess or obliteration of the pouch of Douglas [23], large tumours or locally advanced disease [23, 33, 34], and BMI > 30 kg/m^28^.

Three studies [23, 33, 34] excluded patients with large tumours. Awad et al. [23] specified a size cut-off of > 6 cm. Kim et al. [33] and Park et al. [34] excluded patients based on clinical judgement of specimen size. Zhang et al. [28] used the 2019 International Consensus Guidelines on Colorectal NOSE Surgery [10] to determine patient suitability for transvaginal extraction. This consensus excludes locally advanced tumours, T4 tumours, and advocates a maximum specimen size and BMI cut-off of 5 cm and 35 kg/m^2^ respectively [10].

### Risk of bias, publication of bias, and heterogeneity

**Fig. 2:**
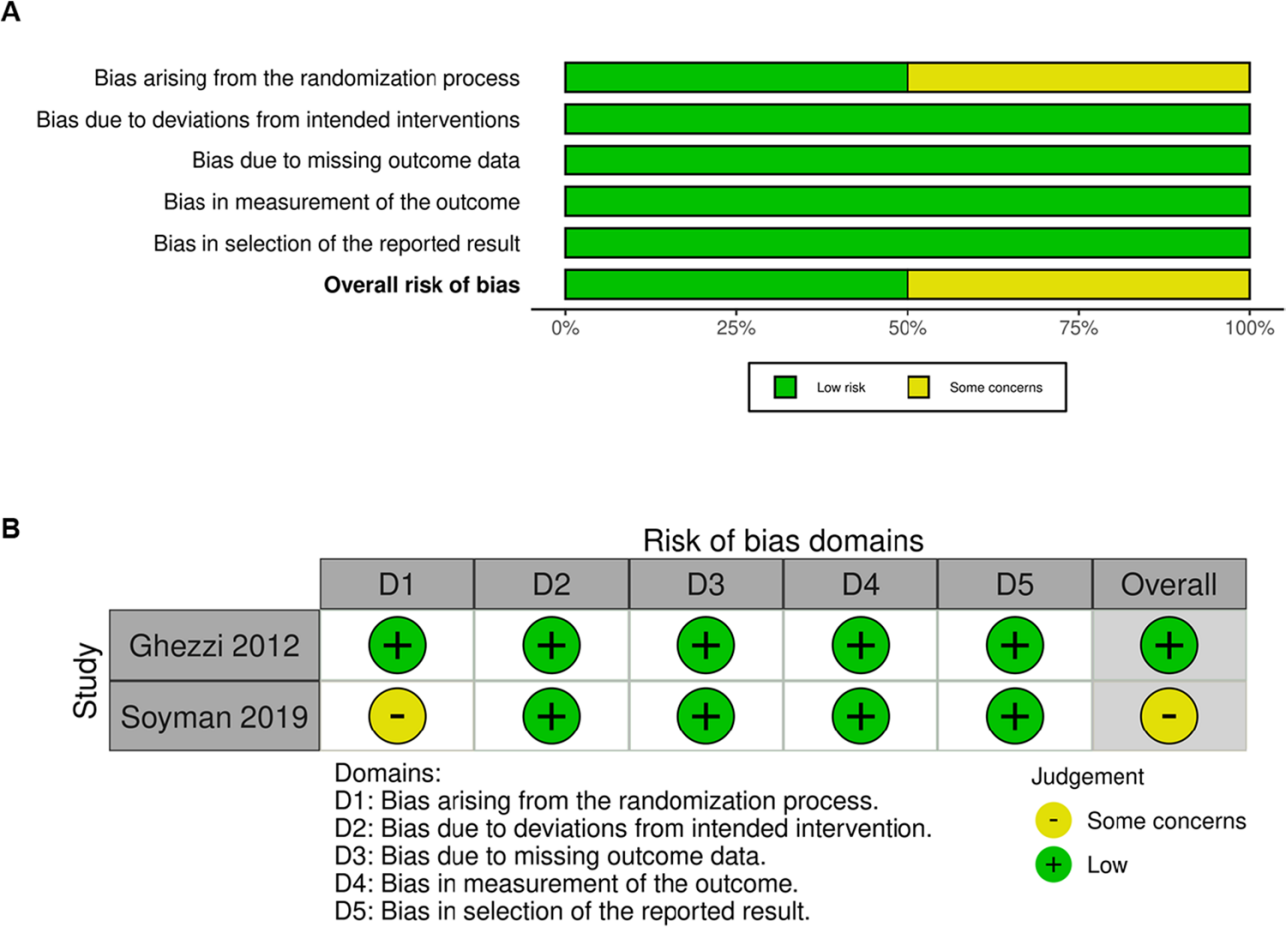
Quality evaluation of RCTs. Assessment of the RoB based on RoB-2 criteria. (**A**) RoB summary. (**B**) RoB traffic-light plot. RoB, risk of bias

**Fig. 3:**
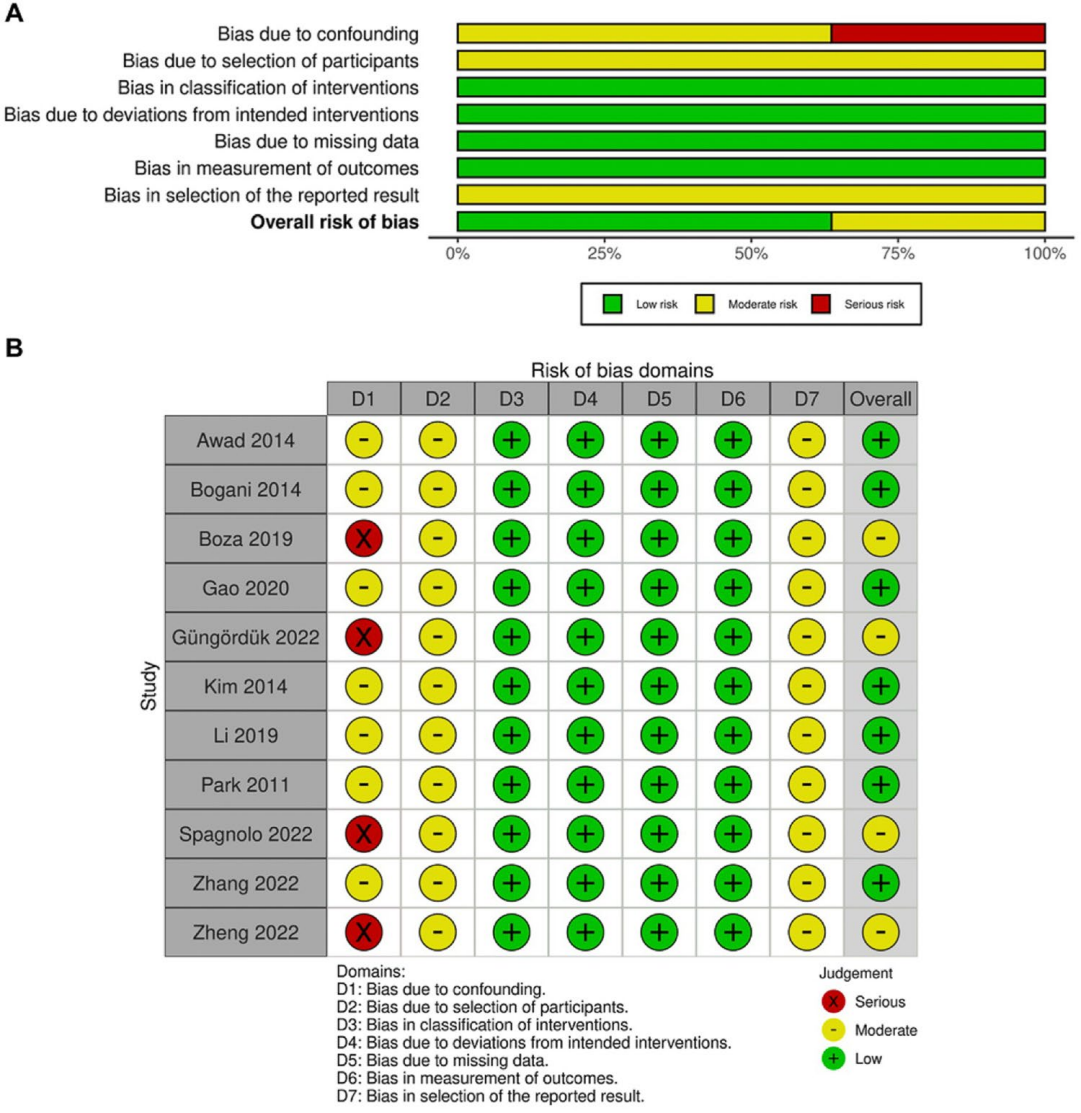
Quality evaluation of non-RCTs. Assessment of the RoB based on ROBINS-I criteria. (**A**) RoB summary. (**B**) RoB traffic-light plot. RoB, risk of bias

RoB assessment for RCTs and non-RCTs are shown in Figs. 2 and 3 respectively. Four retrospective studies [29–31, 35] exhibited bias due to confounding. However, the overall RoB of all included studies was low. Publication bias was deemed low based on the high degree of symmetry in the funnel plot assessment (Supplementary Figure 1).

### Primary outcomes

All outcomes are summarised in Appendix 2.

#### Postoperative day 1 pain

**Fig. 4:**
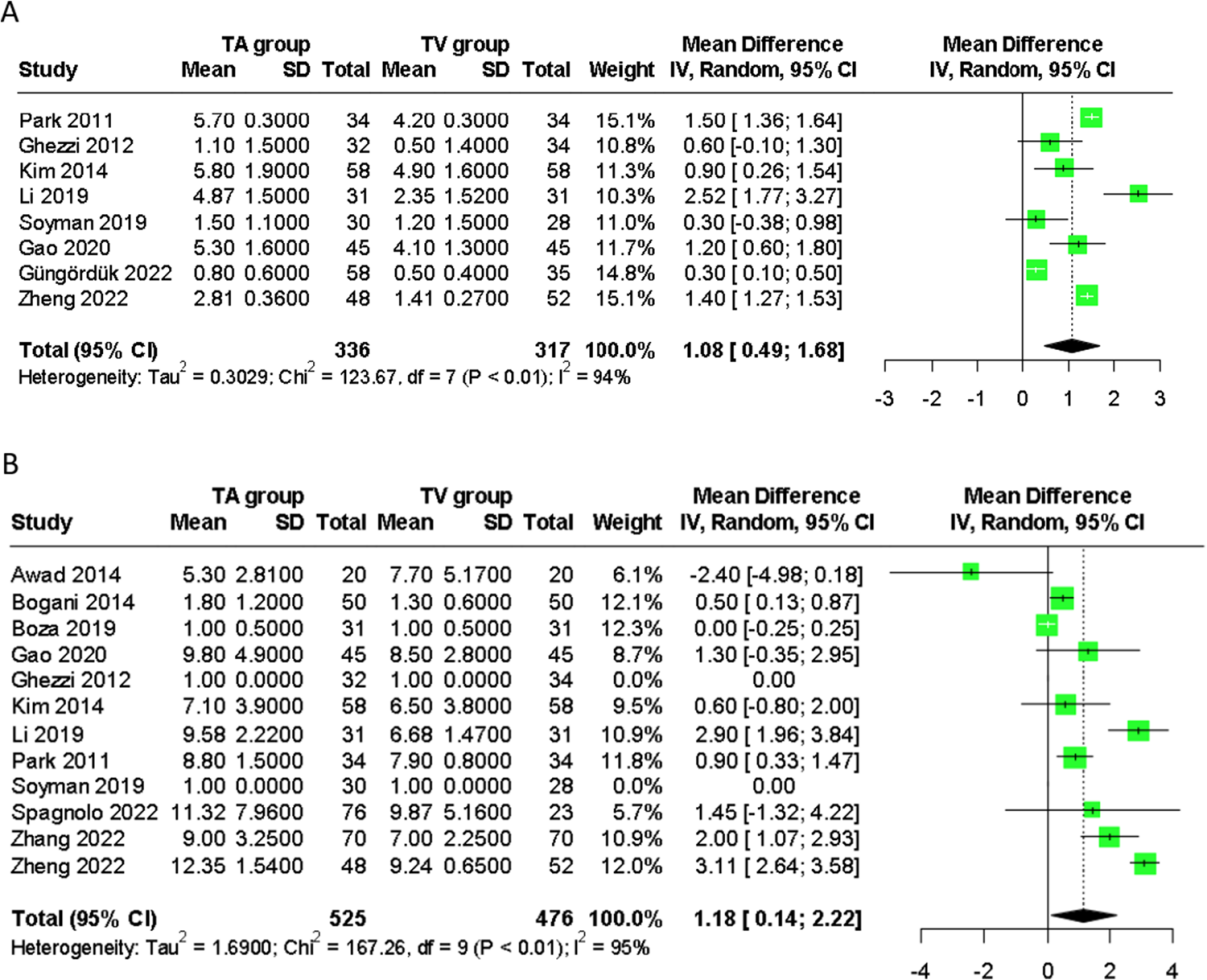
Forest plots comparing (**A**) postoperative day 1 pain score and, (**B**) hospital length of stay following transabdominal and transvaginal specimen extraction in minimally invasive surgery. TA, transabdominal specimen extraction; TV, transvaginal specimen extraction

A meta-analysis of eight studies [25–27, 29, 31–34] including 653 patients, demonstrated a significantly reduced POD1 pain score with TVSE compared to TASE, measured using the visual analogue scale (VAS) (WMD 1.08, 95% CI: 0.49, 1.68) (Fig. 4). Both randomised controlled trials [25, 32] evaluated postoperative pain within the first 24 hours of surgery as primary outcome measures. Ghezzi et al. [25] demonstrated significantly less pain at postoperative 1, 3 and 24 hours with TVSE compared to TASE, while Soyman et al. [32] reported a similar advantage with TVSE at 6 and 24 hours. Park et al. [34] and Li et al. [27] assessed POD 3 and POD 3 and 5 pain respectively, demonstrating less pain with TVSE even at later time points beyond the first postoperative day. Awad et al. [23] reported no significant differences between pain scores on POD 1, 2 or 14.

#### Length of stay

A meta-analysis of 12 studies [23–30, 32–35] reporting this outcome, comprising 1001 patients, showed a significant decrease in LOS with TVSE compared to TASE (WMD 1.18 days, 95% CI: 0.14, 2.22) (Fig. 4).

### Secondary outcomes

#### Operative time

**Fig. 5:**
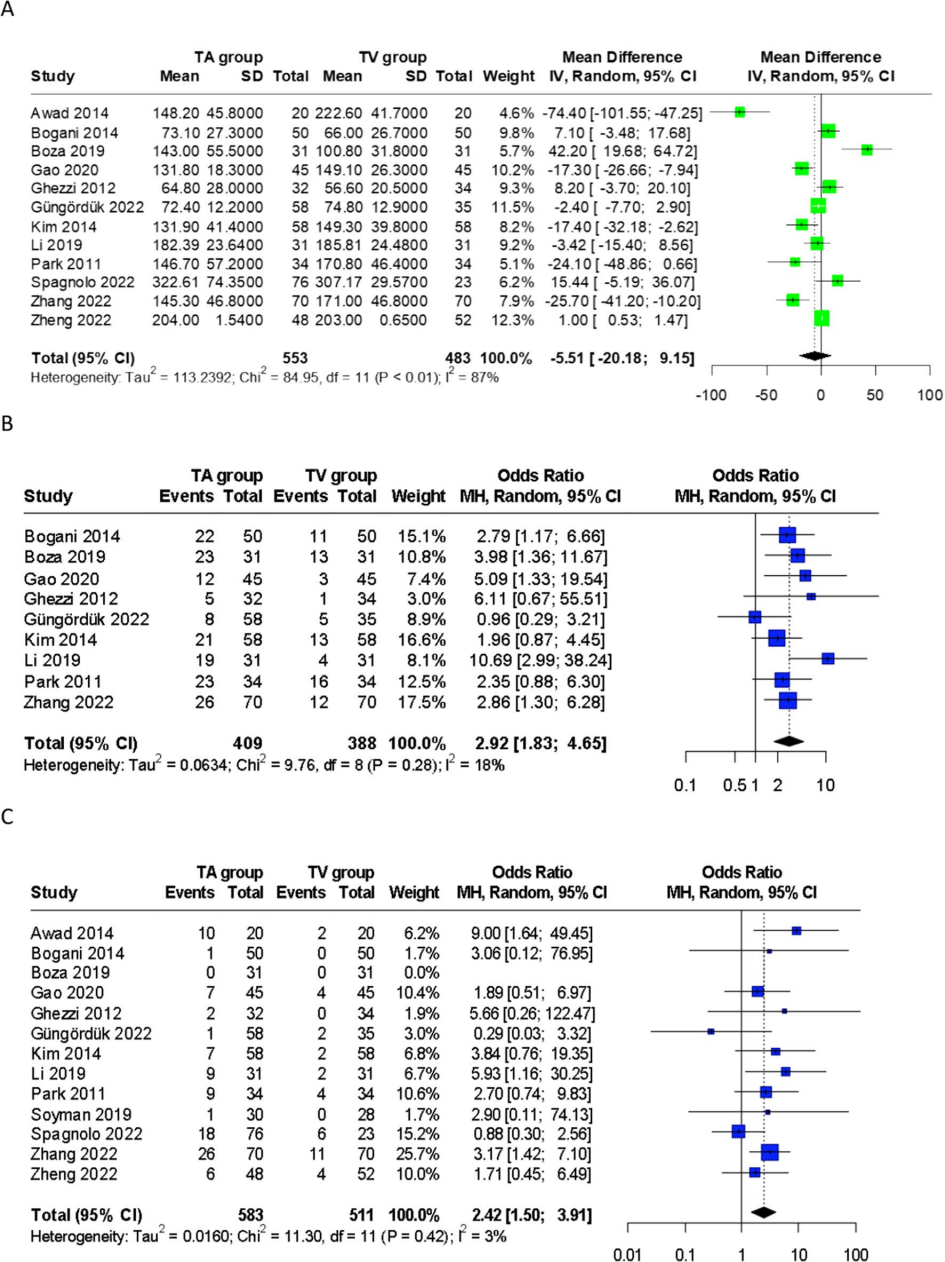

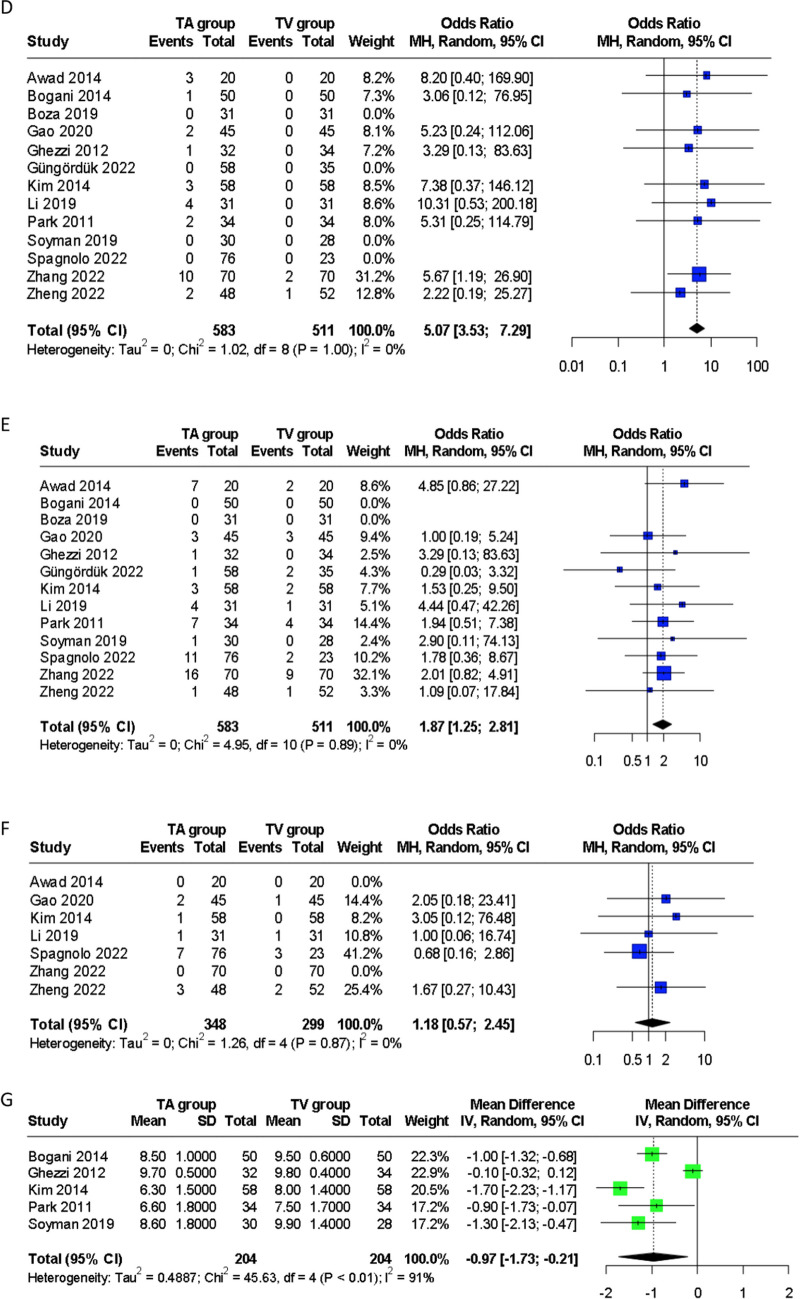
Forest plots of (**A**) Operative duration, (**B**) use of postoperative rescue analgesia, (**C**) overall postoperative morbidity, (**D**) wound-related morbidity, (**E**) non-wound related morbidity, (**F**) anastomotic complications, and (**G**) cosmetic outcomes. TA, transabdominal specimen extraction; TV, transvaginal specimen extraction

Pooled analysis of data from 12 studies [23–31, 33–35], including 1036 patients, showed no significant difference in mean operating time (MD -5.51min, 95% CI: -20.18,9.15) (Fig. 5). Only one study by Awad et al. [23] reported a significantly longer median duration of surgery for TVSE compared to TASE (MD -74.40min, 95% CI -101.55, -47.25). However, this was attributed by the authors to the learning curve required for TVSE surgery, demonstrated by a reduction in operative duration over time as more surgeries were performed.

Boza et al. [30] was the only study to show a significantly quicker surgery time for TVSE versus TASE (MD 42.2min, 95% CI 19.68,62.72). This was attributed to the TASE cohort having larger myomas, requiring morcellation with a power morcellator.

#### Postoperative rescue analgesia

Ten studies [24–28, 30–34] reported the use of additional or rescue analgesia, reflecting the degree of post-surgical pain. Nine studies [24–28, 30, 31, 33, 34] documented the number of patients requiring rescue analgesia, while only one [32] stated the exact analgesic and dosages administered. Pooled data from the nine studies [24–28, 30, 31, 33, 34] inclusive of 797 patients showed significantly fewer patients requiring rescue analgesia in the TVSE group (OR 2.92; 95% CI: 1.83; 4.65) (Fig. 5).

#### Postoperative morbidity

All 13 studies reported postoperative complications [23–35], with the TV group demonstrating a significantly lower overall complication rate compared to TASE (OR 2.42, 95% CI: 1.50; 3.91) (Fig. 5).

Patients who underwent TVSE experienced significantly less wound-related morbidity (OR 5.07, 95% CI: 3.53; 7.29). Moreover, non-wound related morbidity, including ileus, postoperative urinary retention, etc, was also reduced in the TVSE group (OR 1.87, 95% CI: 1.25; 2.81) (Fig. 5). We attributed these findings to be a consequence of reduced postoperative pain, decreased opioid administration, and earlier mobilisation following TVSE compared to TASE. This is consistent with several studies reporting a reduced proportion of postoperative ileus with TVSE [23, 26, 33, 34]. Furthermore, six studies [23, 26–29, 33] also documented faster recovery of gastrointestinal function with TVSE, with a quicker time to oral feeding or passage of gas, compared to abdominal extraction. Anastomosis-related complication rates were similar between both two groups (OR 1.18, 95% CI: 0.57; 2.45) (Fig. 5).

Spagnolo et al. [35] evaluated TVSE versus TASE (via suprapubic extraction) amongst patients who underwent colorectal resection for deep pelvic endometriosis following failure of conservative management. The overall postoperative morbidity rate was similar between both groups (26% versus 25%). There were no infective complications amongst TVSE patients compared to 6.6% in the TASE cohort. The rate of rectovaginal fistulation was 8.7% (*n* = 2) following TVSE versus 3.9% (*n* = 3) after TASE. In this study, the postoperative formation of rectovaginal fistulae was a sequela of surgery for vaginal endometriosis rather than the vaginal extraction procedure per se. Moreover, several other patients in the study developed pelvic fistulation elsewhere (Two ureteral fistula in the TVSE cohort and one ureteral fistula in the TASE cohort), without vaginal involvement. No other study reported instances of rectovaginal fistulation, or other vaginal/cervical morbidity, following TVSE.

#### Cosmetic outcomes

Five studies [24, 25, 32–34] presented cosmetic outcomes using a patient-reported visual analogue scale, assessed 3 to 6 months after surgery. Pooled data showed a significantly higher cosmetic VAS score with TVSE compared to TASE (WMD -0.97, 95% CI: -1.73; -0.21) (Fig. 5).

Li et al. [27] reported patient satisfaction on abdominal wall appearance, with the TVSE group having 100% (*n* = 31) satisfaction rate, compared to 22.6% (*n* = 7) in the TASE group. Zhang et al. [28] used the Patient Scar Assessment Questionnaire (PSAQ) to assess cosmesis after surgery, showing a significantly lower median score following TVSE in both the total subscale score (38 versus 43, *p* = 0.000) and the global subscale score (6 versus 8, *p* = 0.000), reflecting better patient satisfaction compared to TASE.

### Sexual, oncological, and technical outcomes

#### Postoperative sexual dysfunction

Eight studies compared postoperative sexual dysfunction [24–26, 28, 29, 31, 33, 34]. Methods used to evaluate sexual dysfunction varied across the studies. Two studies [28, 29] used the Female Sexual Function Index (FSFI), a widely validated scoring system comprising 19 items over six domains: desire, arousal, lubrication, orgasm, satisfaction, and pain. Güngördük et al. [31] used self-reported dyspareunia with Sexual Function Index (SFI). SFI comprises of five parameters: desire, arousal, lubrication, orgasm and satisfaction, with each component scored from 0 to 10. Five studies [24–26, 33, 34] assessed patient self-reported sexual dysfunction and dyspareunia.

Zhang et al. [28] documented no significant differences in FSFI between TVSE and TASE preoperatively, as well as one year postoperatively. Both patient cohorts experienced a significant drop in FSFI following surgery, particularly in the lubrication domain, although there was no significant difference in the extent of deterioration. A univariate logistic regression analysis evaluating patient age, menstrual status, type of surgery, tumour location, TMN stage, and chemotherapy history also showed no significant risk factors for surgery-related sexual dysfunction.

Zheng et al. [29] evaluated preoperative and postoperative FSFI at 1, 2 and 3 months. The TASE group showed similar sexual function scores before and after surgery, with no significant differences at postoperative 1, 2 and 3 months. Following TVSE, sexual function scores decreased significantly at postoperative one month, but subsequently recovered by 3 months to the same level as the TASE group. No reason was postulated by the authors for the early, temporary decrease in sexual function.

Güngördük et al. [31] found no significant differences in postoperative SFI at 3 months (*p* = 0.424). Amongst studies using self-reported sexual dysfunction, four [24, 26, 33, 34] documented no instances of dyspareunia or sexual dysfunction amongst sexually active patients. Awad et al. [23] described one case of dyspareunia amongst 21 patients (4.8%) who underwent transvaginal NOSE following right hemicolectomy, that resolved by one year after surgery. Interestingly, Güngördük et al. [31] showed a significantly higher incidence of dyspareunia with TASE compared to TVSE at postoperative 3 months. The study found TASE to be an independent risk factor for dyspareunia, postulating that sexual function may be worsened by physical perception of the abdominal scar. The randomised controlled trial by Ghezzi et al. [25] showed similar rates of postoperative dyspareunia amongst both groups at two months’ follow-up.

#### Oncological outcomes

Oncological outcomes were reported in all the six studies [26–29, 33, 34] involving malignant colorectal disease. All six found no differences in lymph node harvest between TASE and TVSE, while three [26, 33, 34] showed no differences with respect to mean tumour size, resection margin involvement, tumour differentiation and TMN stage.

Where documented, tumour recurrence and survival rates between TASE and TVSE were statistically comparable. Gao et al. [26], Li et al. [27] and Kim et al. [33] compared 3-year disease free survival rates while Zheng et al. [29] evaluated 2-year disease free survival. Park et al. [34] and Zhang et al. [28] did not specify postoperative follow-up duration. No study specifically reported any instances of abdominal port-site, pelvic or vaginal disease recurrence.

#### Technical outcomes

Blood loss: Nine studies [24–31, 34] compared intraoperative blood loss between TASE and TVSE, with none showing significant differences.

TVSE failure: Three studies documented intraoperative failure of transvaginal extraction, with an overall failure rate of 3.6% (4 out of 112 patients). Awad et al. [23] reported one (5.0%) failure out of 20 patients, for a large 8 cm specimen. Kim et al. [33] recorded one (1.7%) failure out of 58, attributed to a moderately sized tumour with vaginal atrophy. Park et al. [34] reported two (5.9%) failures out of 34 due to inadequate size of posterior colpotomy relative to the specimen.

Of the four patients who failed TVSE, three were converted to TASE, one via a right lower quadrant transverse incision and two via a Pfannenstiel incision. The final route of specimen extraction was not specified for the last patient. There were no specific mentions amongst the individual studies regarding postoperative complications for patients with failed transvaginal specimen removal.

## Discussion

Despite increasing evidence demonstrating the safety, feasibility, and advantages of natural orifice specimen extraction in abdominal surgery, this technique appears currently limited to specialised centres. This may be due to several factors, including unclear patient selection criteria, unfamiliarity with the technique, and concerns about potential morbidity and oncological outcomes [11]. In addition, surgeons may be hesitant to create an additional vaginal incision, away from the primary organ of disease.

The International Alliance of NOSE Surgery recently published two sets of consensus guidelines, for colorectal cancer [10], and gastric cancer [36]. For transvaginal specimen retrieval, these guidelines advocate excluding locally advanced tumours, and young women who have not completed their family, i.e., with the potential for future vaginal delivery. A specimen diameter upper limit of 5 cm, and a BMI of below 30-35 kg/m^2^ was also recommended for TVSE. Nonetheless, surgeons have routinely performed TVSE for larger tumours [11, 37, 38], with a 2015 systematic review by Kayaalp et al. [37] suggesting a specimen size upper limit of 8 cm.

While tumour size is an important factor, in our experience overall specimen diameter, inclusive of the tumour and attached mesentery, relative to pelvic outlet and conduit diameter is the most crucial factor determining success or failure of TVSE [11]. Careful operator assessment of these factors intraoperatively is essential, as repeated and prolonged attempts at NOSE can increase the risk of specimen rupture. Karagul et al. [38] reported a 27% failure rate with NOSE following colorectal resections. This may be a result of suboptimal patient selection or operator learning curves. Our review of comparative trials demonstrates a high success rate of TVSE in experienced centres, with a low risk of conversion to conventional transabdominal extraction. Operative duration and postoperative morbidity for TVSE were similar compared with TASE.

The recommendation against NOSE for locally advanced cancers [10, 36] relates to the larger specimen size as well as a theoretical risk of tumour seeding during specimen retrieval. Nonetheless, reports of pelvic or vaginal recurrence have been exceedingly few in the literature [39]. The result of this review further supports the oncological safety of TVSE. Suitable locally advanced tumours can be considered for TVSE on a case-by-case basis.

Sexual dysfunction from vaginal incision is an important concern with TVSE. The results of our analysis are consistent with previous evidence demonstrating the negligible impact of the procedure on sexual function [40, 41]. Although Zheng et al. [29] reported a short-term reduction in postoperative sexual function scores in the TVSE cohort compared to TASE, sexual function was subsequently comparable to TASE controls at 3 months. Moreover, this finding may have been a consequence of routine advice to avoid penetrative sexual intercourse for a temporary period following transvaginal surgery, instead of a direct effect of TVSE per se.

Evidence from studies on gynaecological VNOTES (both transvaginal tissue dissection and specimen extraction without abdominal incision) involving the same vaginal incision as TVSE also do not reveal any increased risk to future pregnancies [40]. This suggests that vaginal extraction techniques can be safely applied to selected younger women for non-gynaecological procedures, contrary to the prevailing concerns reflected in current guidelines for colorectal and gastric NOSE surgery.

Only studies on colorectal and gynaecological surgery without concomitant hysterectomy were included in this review based on inclusion criteria. Nonetheless, TVSE has been performed for other intraabdominal and retroperitoneal organs, albeit in non-comparative feasibility studies. Hwang et al. [42] demonstrated the viability of TVSE for the minimally invasive removal of 33 kidneys, 2 livers, 1 stomach, 1 adrenal gland and 1 bladder. The mean time for the TV NOSE procedure in this series was 28 minutes, with an acceptable mean postoperative FSFI sexual functioning score. A 2016 review of TVSE by Kallidonis et al. [40] included studies on TVSE for radical nephrectomies, donor nephrectomies, appendicectomies, cholecystectomies, and distal pancreatectomies, with several studies describing surgical dissection and tissue mobilisation via the vagina prior to transvaginal extraction. A single case of TVSE following combined right hemicolectomy for caecal adenocarcinoma with concomitant liver metastasectomy has also been reported [43].

While NOSE refers to the method of specimen retrieval, colorectal NOSE surgery requires modification of several other crucial aspects of surgery. Preparation of the proximal and distal ends of the bowel for the creation of a fully intracorporeal bowel anastomosis can be technically demanding for inexperienced surgeons, with the risks of intraperitoneal sepsis and anastomotic complications [44, 45]. This review demonstrates the comparable postoperative morbidity rates between TVSE and TASE, albeit within tertiary referral centres. It is therefore important to emphasise the necessity for NOSE operators to be highly proficient laparoscopic surgeons employing appropriate patient selection.

### Strengths and limitations

To our knowledge, this is the first systematic review and meta-analysis specifically evaluating TVSE versus TASE in minimally invasive abdominal surgery. Limitations of this study include the small number of randomised controlled trials and the possibility of selection bias in retrospective studies evaluating advanced surgical procedures. Only colorectal and gynaecological surgeries were analysed as the single arm nature of studies involving other specialty procedures precluded their review. In addition, there was significant heterogeneity of the operative indications and procedure types. The current evidence for TVSE supports continued evaluation in prospective studies and expanded indications for other benign and malignant abdominal disease, as well as a critical appraisal of existing guidelines.

## Conclusion

TVSE has several benefits over TASE in minimally invasive abdominal surgery, including less postoperative pain, reduced analgesia use, shorter length of hospital stays, decreased morbidity, and enhanced cosmesis. TVSE does not result in worse sexual dysfunction and does not increase the risk of local recurrence compared to conventional transabdominal extraction.

### Supplementary information

ESM 1 Supplementary Figure 1 Funnel plots of primary and secondary outcomes. The vertical line in the middle of the funnel shows the average effect size. Given that most studies roughly follow the shape delineated by the funnel displayed in the plots, the probability of publication bias is low. (DOCX 135 kb)

## Data Availability

No datasets were generated or analysed during the current study.

## Appendix 1

Full Search Strategy

### PubMed

((((((((((((Natural orifice specimen extraction) OR (Natural orifice specimen retrieval)) OR (Transvaginal natural orifice specimen extraction)) OR (Transvaginal specimen extraction)) OR (Transvaginal specimen retrieval)) OR (Transanal natural orifice specimen extraction)) OR (Transanal specimen extraction)) OR (Transanal specimen retrieval)) OR (Transrectal natural orifice specimen extraction)) OR (Transrectal specimen extraction)) OR (Transrectal specimen retrieval)) OR (Transcolonic natural orifice specimen extraction)) OR (Transcolonic specimen extraction)) OR (Transcolonic specimen retrieval)

### Embase

1. (Natural orifice specimen extraction or Natural orifice specimen retrieval or Transvaginal natural orifice specimen extraction or Transvaginal specimen extraction or Transvaginal specimen retrieval or Transanal natural orifice specimen extraction or Transanal specimen extraction or Transanal specimen retrieval or Transrectal natural orifice specimen extraction or Transrectal specimen extraction or Transrectal specimen retrieval).af.

2. limit 1 to (english language and “remove preprint records”)

### CENTRAL

IDSearch

#1Natural orifice specimen extraction

#2Natural orifice specimen retrieval

#3Transvaginal natural orifice specimen extraction

#4Transvaginal specimen extraction

#5Transvaginal specimen retrieval

#6Transanal natural orifice specimen extraction

#7Transanal specimen extraction

#8Transanal specimen retrieval

#9Transrectal natural orifice specimen extraction

#10Transrectal specimen extraction

#11Transrectal specimen retrieval

#12#1 OR #2 OR #3 OR #4 OR #5 OR #6 OR #7 OR #8 OR #9 OR #10 OR #11

#13#1 OR #2 OR #3 OR #4 OR #5 OR #6 OR #7 OR #8 OR #9 OR #10 OR #11 in Trials

## Appendix 2

### Summary of All Outcomes

#### Postoperative day 1 pain

**Table:**

Author	TA group			TV group		
	Sample size	Mean (VAS)	SD	Sample size	Mean (VAS)	SD
Park et al. [34]	34	5.7	0.3 (Standard error of mean)	34	4.2	0.3 (Standard error of mean)
Ghezzi et al. [25]	32	1.1	1.5	34	0.5	1.4
Kim et al. [33]	58	5.8	1.9	58	4.9	1.6
Li et al. [27]	31	4.87	1.5	31	2.35	1.52
Soyman et al. [32]	30	1.5	1.1	28	1.2	1.5
Gao et al. [26]	45	5.3	1.6	45	4.1	1.3
Güngördük et al. [31]	58	0.8 (0.6–1.0) [median (95% CI)]	35	0.5 (0.3–0.7) [median (95% CI)]		
Zheng et al. [29]	48	2.81	0.36	52	1.41	0.27

#### Length of Stay

**Table:**

Author	TA group			TV group		
	Sample size	Mean (days)	SD	Sample size	Mean (days)	SD
Awad et al. [23]	20	5.3	2.81	20	7.7	5.17
Bogani et al. [24]	50	1.8	1.2	50	1.3	0.6
Boza et al. [30]	31	2 (1-3) [median (range)]		31	2 (1-3) [median (range)]	
Gao et al. [26]	45	9.8	4.9	45	8.5	2.8
Ghezzi et al. [25]	32	1	0	34	1	0
Kim et al. [33]	58	7.1	3.9	58		3.8
Li et al. [27]	31	9.58	2.22	31	6.68	1.47
Park et al. [34]	34	8.8	1.5	34	7.9	0.8
Soyman et al. [32]	30	1	0	28	1	0
Spagnolo et al. [35]	76	11.32	7.96	23	9.87	5.16
Zhang et al. [28]	70	6 (4–17) [median (range)]		70	6 (3–12) [median (range)]	
Zheng et al. [29]	48	12.35	1.54	52	9.24	0.65

#### Operative time

**Table:**

Author	TA group			TV group		
	Sample size	Mean (min)	SD	Sample size	Mean (min)	SD
Awad et al. [23]	20	148.2	(range 75-258)	20	222.6	(range 136.2-303)
Bogani et al. [24]	50	73.1	27.3	50	66	26.7
Boza et al. [30]	31	110 (65-287) [median (range)]		31	90 (48-175) [median (range)]	
Gao et al. [26]	45	131.8	18.3	45	149.1	26.3
Ghezzi et al. [25]	32	64.8	28	34	56.6	20.5
Güngördük et al. [31]	58	72.4	12.2	35	74.8	12.9
Kim et al. [33]	58	131.9	41.4	58	149.3	39.8
Li et al. [27]	31	182.39	23.64	31	185.81	24.48
Park et al. [34]	34	146.7	57.2	34	170.8	46.4
Spagnolo et al. [35]	76	322.61	74.35	23	307.17	29.57
Zhang et al. [28]	70	137 (60–247) [median (range)]		70	159.5 (89–276) [median (range)]	
Zheng et al. [29]	48	204	1.54	52	203	0.65

#### Postoperative rescue analgesia

**Table:**

Author	TA group		TV group	
	Sample size	n	Sample size	n
Bogani et al. [24]	50	22	50	11
Boza et al. [30]	31	23	31	13
Gao et al. [26]	45	12	45	3
Ghezzi et al. [25]	32	5	34	1
Güngördük et al. [31]	58	8	35	5
Kim et al. [33]	58	21	58	13
Li et al. [27]	31	19	31	4
Park et al. [34]	34	23	34	16
Zhang et al. [28]	70	26	70	12

#### Postoperative morbidity: complication rate and types

**Table:**

Author	TA group					TV group					
	Sample size	n (total)	Wound-related	Anastomosis-related	Others	Sample size	n (total)	Wound-related	Anastomosis-related	TVSE-related	Others
Awad et al. [23]	20	10	3 (2 incisional hernia, 1 surgical site infection)	0	7 (ileus)	20	2	0	0	0	2 (1 postoperative dyspareunia that resolved 1 year after surgery, 1 minimal postoperative vaginal discharge starting 1 week after surgery, resolved in 6 months.
Bogani et al. [24]	50	1*	1 (morcellator trocar site bleeding requiring re-suture of the trocar wound on POD1)	NA	0	50	0*	0	NA	0	0
Boza et al. [30]	31	0	0	NA	0	31	0	0	NA	0	0
Gao et al. [26]	45	7	2 (2 wound infections)	2 (2 anastomotic leaks)	3	45	4	0	1 (anastomotic leak)	0	3 (2 pulmonary infections, 1 intra-abdominal abscess)
Ghezzi et al. [25]	32	2	1 (3cm hematoma at site of a 3mm ancillary port that required readmission for IV antibiotics)	NA	1 (haemoperitoneum managed conservatively)	34	0	0	NA	0	0
Güngördük et al. [31]	58	1	0	NA	1 (post-op fever)	35	2	0	NA	0	2 (2 post-op fever)
Kim et al. [33]	58	7	3 (3 wound infections)	1 (anastomotic leak)	3 (1 ileus, 1 intra-abdominal abscess, 1 post-op bleeding requiring transfusion)	58	2	0	0	0	2 (1 intra-abdominal abscess, 1 post-op bleeding requiring transfusion)
Li et al. [27]	31	9	4 (abdominal wall incision infection or poor healing)	1 (anastomotic fistula)	4 (1 intestinal obstruction, 1 urinary retention, 1 venous thrombosis, 1 pulmonary infection)	31	2	0	1 (anastomotic bleeding)	0	1 (pulmonary infection)
Park et al. [34]	34	9	2	NA	7 (2 haemorrhage requiring transfusion, 1 intra-abdominal abscess, 2 pulmonary infection, 2 ileus)	34	4	0	0	0	4 (2 haemorrhage requiring transfusion, 1 ileus, 1 urinary retention)
Soyman et al*.* [32]	30	1	0	NA	1 (haemoperitoneum managed conservatively)	28	0	0	NA	0	0
Spagnolo et al. [35]	76	18	0	7 (3 rectovaginal fistula, 3 anastomotic dehiscence, 1 ulcer over granuloma over anastomosis requiring antibiotics)	11 (1 Intestinal obstruction, 1 Hematoma in the anterior rectum, 1 Parauterine and presacral abscess treated with antibiotics, 1 Hemotransfusion, 1 Acute urinary retention, 2 Acute pyelonephritis treated with antibiotics, 2 Hemoperitoneum required reoperation, 1 Ureteral fistula required ureteral reimplantation, 1 Peritonitis and vaginal necrosis requiring reoperation)	23	6	0	3 (2 rectovaginal fistula, 1 anastomotic ischemia and stenosis requiring operation)	1 (ureterovaginal fistula requiring operation)	2 (1 hematoma in anterior rectum, 1 ureteral fistula requiring ureteral reimplantation)
Zhang et al. [28]	70	26	10 (9 wound complications, 1 incisional hernia)	0	16	70	11	2	0	0	9
Zheng et al. [29]	48	6	2 (1 incision infection, 1 incisional hernia)	3 (3 anastomotic leak)	1 (abdominal bleeding)	52	4	1 (incision infection)	2 (2 anastomotic leak)	0	1 (abdominal bleeding)

*Only Accordion Grade 3 and above recorded

#### Cosmetic outcomes

**Table:**

Author	TA group			TV group		
	Cosmetic VAS					
	Sample size	Mean (VAS)	SD	Sample size	Mean (VAS)	SD
Bogani et al. [24]	50	8.5	1	50	9.5	0.6
Ghezzi et al. [25]	32	9.7	0.5	34	9.8	0.4
Kim et al. [33]	58	6.3	1.5	58	8	1.4
Park et al. [34]	34	6.6	1.8	34	7.5	1.7
Soyman et al. [32]	30	8.6	1.8	28	9.9	1.4
	Psychometric Scar Assessment Questionnaire (PSAQ)					
	Sample size	n	PSAQ (at 3 months postop)	Sample size	n	PSAQ (at 3 months postop)
Zhang et al. [28]	70	65	total subscale score 38 (34–55), appearance subscale 11 (9–20), symptoms scale 6 (6–11), global subscale score 6 (5–11)	70	68	total subscale score 38 (34–55), appearance subscale 11 (9–20), symptoms scale 6 (6–11), global subscale score 6 (5–11)

#### Postoperative sexual dysfunction

**Table:**

Author	TA group		TV group	
	Postoperative Female Sexual Function Index (FSFI)			
	Sample size	Result	Sample size	Result
Zhang et al. [28]	70	27.3 (13.5–34.2) [median (range)]	70	26.0 (8.8–36.0) [median (range)]
Zheng et al. [29]	48	Presented no fluctuations at 1, 2, and 3 months after operation	52	One month after surgery, the above indexes of the observation group decreased significantly and then gradually recovered to the preoperative level
	Sexual Function Index (SFI)			
Güngördük et al. [31]	58	31.5 (30.4–32.7) [Median (95% CI)]	35	30.8 (29.3–32.3) [Median (95% CI)]

### Blood loss

**Table:**

Author	TA group			TV group		
	Sample size	Mean (ml)	SD	Sample size	Mean (ml)	SD
Bogani et al. [24]	50	100 (50–900) [median (range)]		50	100 (50–700) [median (range)]	
Boza et al. [30]	31	100 (10-1000) [median (range)]		31	125 (20-300) [median (range)]	
Gao et al. [26]	45	79.5	43.7	45	64.9	36.5
Ghezzi et al. [25]	32	10 (10-50) [median (range)]		34	10 (10-200) [median (range)]	
Güngördük et al. [31]	58	10.5	4.8	35	8.8	4.2
Li et al. [27]	31	68.39	45.61	31	62.26	46.88
Park et al. [34]	34	32.3	32.6	34	42.5	34.9
Zhang et al. [28]	70	20 (10–600) [median (range)]		70	20 (5–120) [median (range)]	
Zheng et al. [29]	48	100	3.22	52	85	1.26
