# Supplementary material for: Transvaginal versus transabdominal specimen extraction in minimally invasive surgery: a systematic review and meta-analysis
Source: Langenbecks Arch Surg. 2024 Jun 3;409(1):172. doi: 10.1007/s00423-024-03361-5 (PMC11147895; doi:10.1007/s00423-024-03361-5)

# Appendix 1

Full Search Strategy

**PubMed**

((((((((((((Natural orifice specimen extraction) OR (Natural orifice specimen retrieval)) OR (Transvaginal natural orifice specimen extraction)) OR (Transvaginal specimen extraction)) OR (Transvaginal specimen retrieval)) OR (Transanal natural orifice specimen extraction)) OR (Transanal specimen extraction)) OR (Transanal specimen retrieval)) OR (Transrectal natural orifice specimen extraction)) OR (Transrectal specimen extraction)) OR (Transrectal specimen retrieval)) OR (Transcolonic natural orifice specimen extraction)) OR (Transcolonic specimen extraction)) OR (Transcolonic specimen retrieval)

**Embase**

1. (Natural orifice specimen extraction or Natural orifice specimen retrieval or Transvaginal natural orifice specimen extraction or Transvaginal specimen extraction or Transvaginal specimen retrieval or Transanal natural orifice specimen extraction or Transanal specimen extraction or Transanal specimen retrieval or Transrectal natural orifice specimen extraction or Transrectal specimen extraction or Transrectal specimen retrieval).af.

2. limit 1 to (english language and "remove preprint records")

**CENTRAL**

ID Search

#1 Natural orifice specimen extraction

#2 Natural orifice specimen retrieval

#3 Transvaginal natural orifice specimen extraction

#4 Transvaginal specimen extraction

#5 Transvaginal specimen retrieval

#6 Transanal natural orifice specimen extraction

#7 Transanal specimen extraction

#8 Transanal specimen retrieval

#9 Transrectal natural orifice specimen extraction

#10 Transrectal specimen extraction

#11 Transrectal specimen retrieval

#12 #1 OR #2 OR #3 OR #4 OR #5 OR #6 OR #7 OR #8 OR #9 OR #10 OR #11

#13 #1 OR #2 OR #3 OR #4 OR #5 OR #6 OR #7 OR #8 OR #9 OR #10 OR #11 in Trials

# Appendix 2

Summary of All Outcomes.

Postoperative day 1 pain

| Author | TA group | | | TV group | | |
| --- | --- | --- | --- | --- | --- | --- |
|  | Sample size | Mean (VAS) | SD | Sample size | Mean (VAS) | SD |
| Park *et al.*[^34^](#_ENREF_34) | 34 | 5.7 | 0.3 (Standard error of mean) | 34 | 4.2 | 0.3 (Standard error of mean) |
| Ghezzi *et al.*[^25^](#_ENREF_25) | 32 | 1.1 | 1.5 | 34 | 0.5 | 1.4 |
| Kim *et al.*[^33^](#_ENREF_33) | 58 | 5.8 | 1.9 | 58 | 4.9 | 1.6 |
| Li *et al.*[^27^](#_ENREF_27) | 31 | 4.87 | 1.5 | 31 | 2.35 | 1.52 |
| Soyman *et al.*[^32^](#_ENREF_32) | 30 | 1.5 | 1.1 | 28 | 1.2 | 1.5 |
| Gao *et al.*[^26^](#_ENREF_26) | 45 | 5.3 | 1.6 | 45 | 4.1 | 1.3 |
| Güngördük *et al.*[^31^](#_ENREF_31) | 58 | 0.8 (0.6–1.0) [median (95% CI)] | 35 | 0.5 (0.3–0.7) [median (95% CI)] |  |  |
| Zheng *et al.*[^29^](#_ENREF_29) | 48 | 2.81 | 0.36 | 52 | 1.41 | 0.27 |

Length of Stay

| Author | TA group | | | TV group | | |
| --- | --- | --- | --- | --- | --- | --- |
|  | Sample size | Mean (days) | SD | Sample size | Mean (days) | SD |
| Awad *et al.*[^23^](#_ENREF_23) | 20 | 5.3 | 2.81 | 20 | 7.7 | 5.17 |
| Bogani *et al.*[^24^](#_ENREF_24) | 50 | 1.8 | 1.2 | 50 | 1.3 | 0.6 |
| Boza *et al.*[^30^](#_ENREF_30) | 31 | 2 (1-3) [median (range)] | | 31 | 2 (1-3) [median (range)] | |
| Gao *et al.*[^26^](#_ENREF_26) | 45 | 9.8 | 4.9 | 45 | 8.5 | 2.8 |
| Ghezzi *et al.*[^25^](#_ENREF_25) | 32 | 1 | 0 | 34 | 1 | 0 |
| Kim *et al.*[^33^](#_ENREF_33) | 58 | 7.1 | 3.9 | 58 |  | 3.8 |
| Li *et al.*[^27^](#_ENREF_27) | 31 | 9.58 | 2.22 | 31 | 6.68 | 1.47 |
| Park *et al.*[^34^](#_ENREF_34) | 34 | 8.8 | 1.5 | 34 | 7.9 | 0.8 |
| Soyman *et al.*[^32^](#_ENREF_32) | 30 | 1 | 0 | 28 | 1 | 0 |
| Spagnolo *et al.*[^35^](#_ENREF_35) | 76 | 11.32 | 7.96 | 23 | 9.87 | 5.16 |
| Zhang *et al.*[^28^](#_ENREF_28) | 70 | 6 (4–17) [median (range)] | | 70 | 6 (3–12) [median (range)] | |
| Zheng *et al.*[^29^](#_ENREF_29) | 48 | 12.35 | 1.54 | 52 | 9.24 | 0.65 |

Operative time

| Author | TA group | | | TV group | | |
| --- | --- | --- | --- | --- | --- | --- |
|  | Sample size | Mean (min) | SD | Sample size | Mean (min) | SD |
| Awad et al.[^23^](#_ENREF_23) | 20 | 148.2 | (range 75-258) | 20 | 222.6 | (range 136.2-303) |
| Bogani et al.[^24^](#_ENREF_24) | 50 | 73.1 | 27.3 | 50 | 66 | 26.7 |
| Boza et al.[^30^](#_ENREF_30) | 31 | 110 (65-287) [median (range)] | | 31 | 90 (48-175) [median (range)] | |
| Gao et al.[^26^](#_ENREF_26) | 45 | 131.8 | 18.3 | 45 | 149.1 | 26.3 |
| Ghezzi et al.[^25^](#_ENREF_25) | 32 | 64.8 | 28 | 34 | 56.6 | 20.5 |
| Güngördük et al.[^31^](#_ENREF_31) | 58 | 72.4 | 12.2 | 35 | 74.8 | 12.9 |
| Kim et al.[^33^](#_ENREF_33) | 58 | 131.9 | 41.4 | 58 | 149.3 | 39.8 |
| Li et al.[^27^](#_ENREF_27) | 31 | 182.39 | 23.64 | 31 | 185.81 | 24.48 |
| Park et al.[^34^](#_ENREF_34) | 34 | 146.7 | 57.2 | 34 | 170.8 | 46.4 |
| Spagnolo et al.[^35^](#_ENREF_35) | 76 | 322.61 | 74.35 | 23 | 307.17 | 29.57 |
| Zhang et al.[^28^](#_ENREF_28) | 70 | 137 (60–247) [median (range)] | | 70 | 159.5 (89–276) [median (range)] | |
| Zheng et al.[^29^](#_ENREF_29) | 48 | 204 | 1.54 | 52 | 203 | 0.65 |

Postoperative rescue analgesia

| Author | TA group | | TV group | |
| --- | --- | --- | --- | --- |
|  | Sample size | n | Sample size | n |
| Bogani et al.[^24^](#_ENREF_24) | 50 | 22 | 50 | 11 |
| Boza et al.[^30^](#_ENREF_30) | 31 | 23 | 31 | 13 |
| Gao et al.[^26^](#_ENREF_26) | 45 | 12 | 45 | 3 |
| Ghezzi et al.[^25^](#_ENREF_25) | 32 | 5 | 34 | 1 |
| Güngördük et al.[^31^](#_ENREF_31) | 58 | 8 | 35 | 5 |
| Kim et al.[^33^](#_ENREF_33) | 58 | 21 | 58 | 13 |
| Li et al.[^27^](#_ENREF_27) | 31 | 19 | 31 | 4 |
| Park et al.[^34^](#_ENREF_34) | 34 | 23 | 34 | 16 |
| Zhang *et al.*[^28^](#_ENREF_28) | 70 | 26 | 70 | 12 |

Postoperative morbidity: complication rate and types

| Author | TA group | | | | | TV group | | | | | |
| --- | --- | --- | --- | --- | --- | --- | --- | --- | --- | --- | --- |
|  | Sample size | n (total) | Wound-related | Anastomosis-related | Others | Sample size | n (total) | Wound-related | Anastomosis-related | TVSE-related | Others |
| Awad et al.[^23^](#_ENREF_23) | 20 | 10 | 3 (2 incisional hernia, 1 surgical site infection) | 0 | 7 (ileus) | 20 | 2 | 0 | 0 | 0 | 2 (1 postoperative dyspareunia that resolved 1 year after surgery, 1 minimal postoperative vaginal discharge starting 1 week after surgery, resolved in 6 months. |
| Bogani et al.[^24^](#_ENREF_24) | 50 | 1* | 1 (morcellator trocar site bleeding requiring re-suture of the trocar wound on POD1) | NA | 0 | 50 | 0* | 0 | NA | 0 | 0 |
| Boza et al.[^30^](#_ENREF_30) | 31 | 0 | 0 | NA | 0 | 31 | 0 | 0 | NA | 0 | 0 |
| Gao et al.[^26^](#_ENREF_26) | 45 | 7 | 2 (2 wound infections) | 2 (2 anastomotic leaks) | 3 | 45 | 4 | 0 | 1 (anastomotic leak) | 0 | 3 (2 pulmonary infections, 1 intra-abdominal abscess) |
| Ghezzi et al.[^25^](#_ENREF_25) | 32 | 2 | 1 (3cm hematoma at site of a 3mm ancillary port that required readmission for IV antibiotics) | NA | 1 (haemoperitoneum managed conservatively) | 34 | 0 | 0 | NA | 0 | 0 |
| Güngördük et al.[^31^](#_ENREF_31) | 58 | 1 | 0 | NA | 1 (post-op fever) | 35 | 2 | 0 | NA | 0 | 2 (2 post-op fever) |
| Kim et al.[^33^](#_ENREF_33) | 58 | 7 | 3 (3 wound infections) | 1 (anastomotic leak) | 3 (1 ileus, 1 intra-abdominal abscess, 1 post-op bleeding requiring transfusion) | 58 | 2 | 0 | 0 | 0 | 2 (1 intra-abdominal abscess, 1 post-op bleeding requiring transfusion) |
| Li et al.[^27^](#_ENREF_27) | 31 | 9 | 4 (abdominal wall incision infection or poor healing) | 1 (anastomotic fistula) | 4 (1 intestinal obstruction, 1 urinary retention, 1 venous thrombosis, 1 pulmonary infection) | 31 | 2 | 0 | 1 (anastomotic bleeding) | 0 | 1 (pulmonary infection) |
| Park et al.[^34^](#_ENREF_34) | 34 | 9 | 2 | NA | 7 (2 haemorrhage requiring transfusion, 1 intra-abdominal abscess, 2 pulmonary infection, 2 ileus) | 34 | 4 | 0 | 0 | 0 | 4 (2 haemorrhage requiring transfusion, 1 ileus, 1 urinary retention) |
| Soyman *et al.*[^32^](#_ENREF_32) | 30 | 1 | 0 | NA | 1 (haemoperitoneum managed conservatively) | 28 | 0 | 0 | NA | 0 | 0 |
| Spagnolo et al.[^35^](#_ENREF_35) | 76 | 18 | 0 | 7 (3 rectovaginal fistula, 3 anastomotic dehiscence, 1 ulcer over granuloma over anastomosis requiring antibiotics) | 11 (1 Intestinal obstruction, 1 Hematoma in the anterior rectum, 1 Parauterine and presacral abscess treated with antibiotics, 1 Hemotransfusion, 1 Acute urinary retention, 2 Acute pyelonephritis treated with antibiotics, 2 Hemoperitoneum required reoperation, 1 Ureteral fistula required ureteral reimplantation, 1 Peritonitis and vaginal necrosis requiring reoperation) | 23 | 6 | 0 | 3 (2 rectovaginal fistula, 1 anastomotic ischemia and stenosis requiring operation) | 1 (ureterovaginal fistula requiring operation) | 2 (1 hematoma in anterior rectum, 1 ureteral fistula requiring ureteral reimplantation) |
| Zhang et al.[^28^](#_ENREF_28) | 70 | 26 | 10 (9 wound complications, 1 incisional hernia) | 0 | 16 | 70 | 11 | 2 | 0 | 0 | 9 |
| Zheng et al.[^29^](#_ENREF_29) | 48 | 6 | 2 (1 incision infection, 1 incisional hernia) | 3 (3 anastomotic leak) | 1 (abdominal bleeding) | 52 | 4 | 1 (incision infection) | 2 (2 anastomotic leak) | 0 | 1 (abdominal bleeding) |

*Only Accordion Grade 3 and above recorded

Cosmetic outcomes

| Author | TA group | | | TV group | | |
| --- | --- | --- | --- | --- | --- | --- |
|  | **Cosmetic VAS** | | | | | |
|  | Sample size | Mean (VAS) | SD | Sample size | Mean (VAS) | SD |
| Bogani et al.[^24^](#_ENREF_24) | 50 | 8.5 | 1 | 50 | 9.5 | 0.6 |
| Ghezzi et al.[^25^](#_ENREF_25) | 32 | 9.7 | 0.5 | 34 | 9.8 | 0.4 |
| Kim et al.[^33^](#_ENREF_33) | 58 | 6.3 | 1.5 | 58 | 8 | 1.4 |
| Park et al.[^34^](#_ENREF_34) | 34 | 6.6 | 1.8 | 34 | 7.5 | 1.7 |
| Soyman *et al.*[^32^](#_ENREF_32) | 30 | 8.6 | 1.8 | 28 | 9.9 | 1.4 |
|  | **Psychometric Scar Assessment Questionnaire (PSAQ)** | | | | | |
|  | Sample size | n | PSAQ (at 3 months postop) | Sample size | n | PSAQ (at 3 months postop) |
| Zhang et al.[^28^](#_ENREF_28) | 70 | 65 | total subscale score 38 (34–55), appearance subscale 11 (9–20), symptoms scale 6 (6–11), global subscale score 6 (5–11) | 70 | 68 | total subscale score 38 (34–55), appearance subscale 11 (9–20), symptoms scale 6 (6–11), global subscale score 6 (5–11) |

Postoperative sexual dysfunction

| Author | TA group | | TV group | |
| --- | --- | --- | --- | --- |
|  | **Postoperative Female Sexual Function Index (FSFI)** | | | |
|  | Sample size | Result | Sample size | Result |
| Zhang et al.[^28^](#_ENREF_28) | 70 | 27.3 (13.5–34.2) [median (range)] | 70 | 26.0 (8.8–36.0) [median (range)] |
| Zheng et al.[^29^](#_ENREF_29) | 48 | Presented no fluctuations at 1, 2, and 3 months after operation | 52 | One month after surgery, the above indexes of the observation group decreased significantly and then gradually recovered to the preoperative level |
|  | **Sexual Function Index (SFI)** | | | |
| Güngördük et al.[^31^](#_ENREF_31) | 58 | 31.5 (30.4–32.7) [Median (95% CI)] | 35 | 30.8 (29.3–32.3) [Median (95% CI)] |

Oncological outcomes

Blood loss

| Author | TA group | | | TV group | | |
| --- | --- | --- | --- | --- | --- | --- |
|  | Sample size | Mean (ml) | SD | Sample size | Mean (ml) | SD |
| Bogani *et al.*[^24^](#_ENREF_24) | 50 | 100 (50–900) [median (range)] | | 50 | 100 (50–700) [median (range)] | |
| Boza *et al.*[^30^](#_ENREF_30) | 31 | 100 (10-1000) [median (range)] | | 31 | 125 (20-300) [median (range)] | |
| Gao *et al.*[^26^](#_ENREF_26) | 45 | 79.5 | 43.7 | 45 | 64.9 | 36.5 |
| Ghezzi *et al.*[^25^](#_ENREF_25) | 32 | 10 (10-50) [median (range)] | | 34 | 10 (10-200) [median (range)] | |
| Güngördük *et al.*[^31^](#_ENREF_31) | 58 | 10.5 | 4.8 | 35 | 8.8 | 4.2 |
| Li *et al.*[^27^](#_ENREF_27) | 31 | 68.39 | 45.61 | 31 | 62.26 | 46.88 |
| Park *et al.*[^34^](#_ENREF_34) | 34 | 32.3 | 32.6 | 34 | 42.5 | 34.9 |
| Zhang *et al.*[^28^](#_ENREF_28) | 70 | 20 (10–600) [median (range)] | | 70 | 20 (5–120) [median (range)] | |
| Zheng *et al.*[^29^](#_ENREF_29) | 48 | 100 | 3.22 | 52 | 85 | 1.26 |

# Supplementary Figures

Supplementary Figure 1 Funnel plots of primary and secondary outcomes. The vertical line in the middle of the funnel shows the average effect size. Given that most studies roughly follow the shape delineated by the funnel displayed in the plots, the probability of publication bias is low.


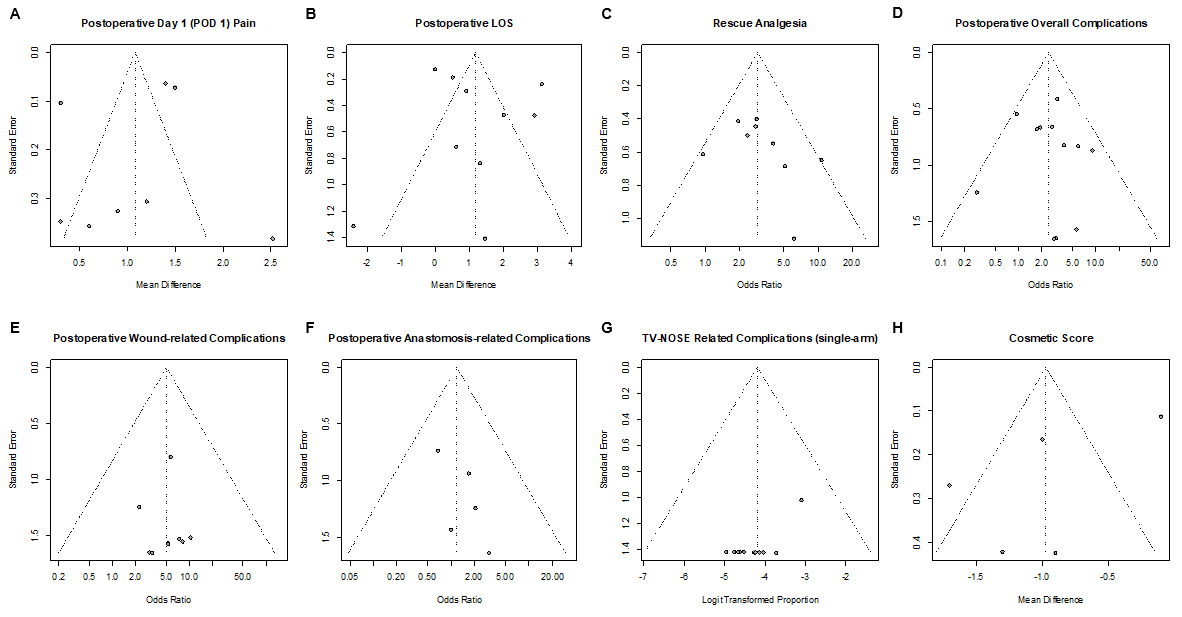

Supplement: Supplementary file 1 — Supplementary Figure 1 Funnel plots of primary and secondary outcomes. The vertical line in the middle of the funnel shows the average effect size. Given that most studies roughly follow the shape delineated by the funnel displayed in the plots, the probability of publication bias is low. (DOCX 135 kb) [file 423_2024_3361_MOESM1_ESM.docx]
